# Multiwall Carbon Nanotube-Induced Apoptosis and Antioxidant Gene Expression in the Gills, Liver, and Intestine of *Oryzias latipes*

**DOI:** 10.1155/2015/485343

**Published:** 2015-06-03

**Authors:** Jin Wuk Lee, Young Chul Choi, Rosa Kim, Sung Kyu Lee

**Affiliations:** ^1^Gyeongnam Department of Environmental Toxicology and Chemistry, Korea Institute of Toxicology, Jin-Ju, Gyeongsangnam-do 660-844, Republic of Korea; ^2^CNT Research Group, R&D Center, Hanwha Chemical Corporation, 76 Gajeong-ro, Yuseong-gu, Daejeon 305-804, Republic of Korea; ^3^Environmental Biology Research Center, Gyeongnam Department of Environmental Toxicology and Chemistry, Korea Institute of Toxicology, Jin-Ju, Gyeongsangnam-do 660-844, Republic of Korea

## Abstract

Multiwall carbon nanotubes (MWCNTs) have many attractive properties with potential applications in various fields. Despite their usefulness, however, the associated waste can be hazardous to the environment. To examine adverse effects in aquatic environments, *Oryzias latipes* were exposed to MWCNTs dispersed in water for 14 days and apoptosis and antioxidant gene expression were observed. This work showed that in gills exposed to 100 mg/L MWCNTs for 4 days, there was significant *p53*, *caspase-3 (Cas3)*, *caspase-8 (Cas8)*, and *caspase-9 (Cas9)* gene expression relative to the controls, while *catalase (CAT)* and *glutathione-S-transferase (GST)* expression were reduced. At 14 days, *CAT*, *GST*, and *metallothionein (MT)* were induced significantly in the gills and *Cas3*, *Cas8*, and *Cas9* were induced in the liver. No significant gene induction was seen in intestine. Intracellular reactive oxygen species (ROS) were increased significantly only at 14 days. Histologically, no apoptosis was observed with exposure to 100 mg/L MWCNTs for 21 days. The gills were more sensitive to MWCNT toxicity than the other organs. Males had higher apoptosis gene induction than females. These results demonstrated that MWCNTs could cause apoptosis in a manner influenced by tissue and gender in aqueous environments.

## 1. Introduction

Aquatic environments are the ultimate sink for many contaminants, via either direct discharge or hydrological and atmospheric processes. Since fish occur in virtually all aquatic environments and play a major role in aquatic food webs, fish are useful for monitoring pollutants in aquatic environments [1].* Oryzias latipes* has been used widely as an experimental fish for monitoring pollutants for over a century [2]. Fish liver is a major detoxification organ and the gills and intestine are useful monitor organs that reflect the health of aquatic organisms and have been used as target organs in aquatic toxicity studies [3–5].

Multiwall carbon nanotubes (MWCNTs) have many attractive electrical, mechanical, and thermal properties with potential applications in engineering, electronics, and medicine [6–8]. Nevertheless, MWCNTs are potentially very harmful environmental pollutants because of three major factors: the shape of the particles; their surface reactivity; and their clearance difficulty [9]. To improve the mechanical properties of MWCNTs, sufficient wetting and uniform dispersion in various media, including aqueous solutions, are required [6, 10]. Therefore, it is possible that wastewater containing dispersed MWCNTs can be introduced into aquatic environments, where they might have hazardous effects.

The previous studies have demonstrated that MWCNTs cause oxidative stress and apoptosis both* in vivo* and* in vitro*. Rainbow trout (*Oncorhynchus mykiss*) exposed to Dietary SWCNT showed significant elevation of TBARS indicative of oxidative stress [11].* Chlorella vulgaris* exposed to MWCNT produced an increase in the level of the antioxidant enzyme superoxide dismutase (SOD) [12]. MWCNT-induced pulmonary toxicity and the expression of proteins related to apoptosis (caspase-3 and caspase-8) were observed on exposing BALB/c mice to aerosolized MWCNTs [9]. Increased apoptosis and caspase-3, caspase-8, and caspase-9 protein activity were observed in RAW 264.7 cells exposed to acid-treated and taurine functionalized MWCNTs [13]. Dose-dependent cytotoxicity in RAW 264.7 macrophages and A549 cells was observed as membrane leakage, liquid peroxidation, activation of the NF-*κ*B signaling pathway, the secretion of cytokines and chemokines, and protein release [8, 14]. Activating caspase-3 and caspase-7 led to MWCNT-induced apoptosis* in vitro* [15]. MWCNTs activated several apoptosis-inducing factors in rat LE cells, including p53, p21, and Bax protein [16]. Although the CNT-induced antioxidant expression and apoptosis occurrence have not been previously observed in aquatic organisms, other nanoparticles caused this kind of response. Zebrafish (*D. rerio*) exposed to silver nanoparticles showed an increase in both* p53*-related proapoptotic genes (e.g.,* Bax*) and parameters of oxidative stress (e.g., malondialdehyde) [17]. Juvenile common carp (*C. carpio*) exposed to TiO_2_ revealed the reduction of antioxidant enzyme activity and apoptosis in hepatocytes and an increase in lipid peroxidation [18]. Copper nanoparticles produced an increase in malondialdehyde levels and in the number of apoptotic cells, but a decrease in SOD activity in juvenile* Epinephelus coioides* [19].

Apoptosis is a programmed cell death pathway that is essential in the development of multicellular organisms and functions in the maintenance of homoeostasis. The abnormal induction of apoptosis can cause various diseases, such as neurodegenerative and cardiovascular diseases [20, 21]. It has been reported that* caspase-3* (*Cas3*),* Cas8*, and* Cas9* gene induction causes apoptosis, and several factors can influence apoptosis and antioxidant gene expression, such as gender and tissue type [13, 22–28].

The exact mechanism of MWCNT-induced ROS production and apoptosis is unclear, although frustrated phagocytosis and the Fenton reaction caused by metal impurities introduced with MWCNTs during their synthesis might play roles [29, 30]. MWCNTs have a high aspect ratio and macrophages cannot lengthen sufficiently to enclose long nanotubes, resulting in incomplete or frustrated phagocytosis [31, 32]. If frustrated phagocytosis occurs, superoxide produced in the macrophage phagosome is released into the extracellular and intracellular space. This superoxide fluxes can lead to oxidative stress, apoptosis, inflammation, and so on [13, 33, 34]. In addition, metals such as iron induce hydroxyl radicals via the Fenton reaction and ROS have been recognized as inducers of apoptosis [35–37].

Although it has been postulated that wastewater containing dispersed MWCNTs can cause abnormal apoptosis and inflammation in aquatic species, only their toxicity was studied [11, 38–42]. Moreover, no investigations were conducted to assess apoptosis by MWCNTs in fish and in other aquatic organism. Therefore, we have investigated the MWCNT-induced antioxidant (*catalase* (*CAT*),* glutathione-S-transferase* (*GST*)),* metallothionein* (*MT*), and apoptosis (*caspase 3* (*Cas3*),* caspase 8* (*Cas8*),* caspase 9* (*Cas9*), and* p53*) gene expression of* Oryzias latipes* in fish in an aquatic environment. In addition, gender, tissue, and temporal differences in gene expression were evaluated.

## 2. Materials and Methods

### 2.1. Chemicals

HCO-40 (PEG-40 hydrogenated castor oil) was received from LG Household & Care Ltd. (Seoul, South Korea). MWCNT was received from Hanwha chemical Co., Ltd. (Seoul, South Korea). The other chemicals used in this study were purchased from Sigma-Aldrich Co. (St. Louis, MO, USA).

### 2.2. MWCNT Dispersion and the Characterization of MWCNTs

**Figure 1:**
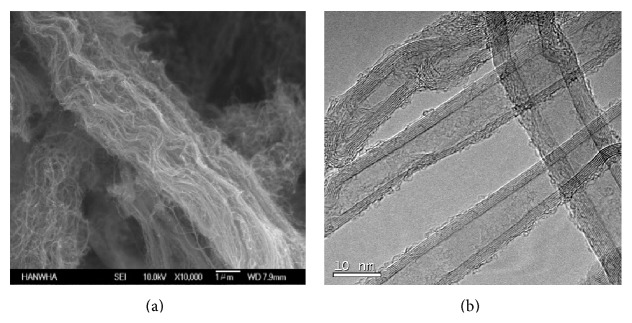
TEM and SEM image. (a) The morphology of the MWCNTs was captured using scanning electron microscopy image and (b) transmission electron microscopy image.

**Figure 2:**
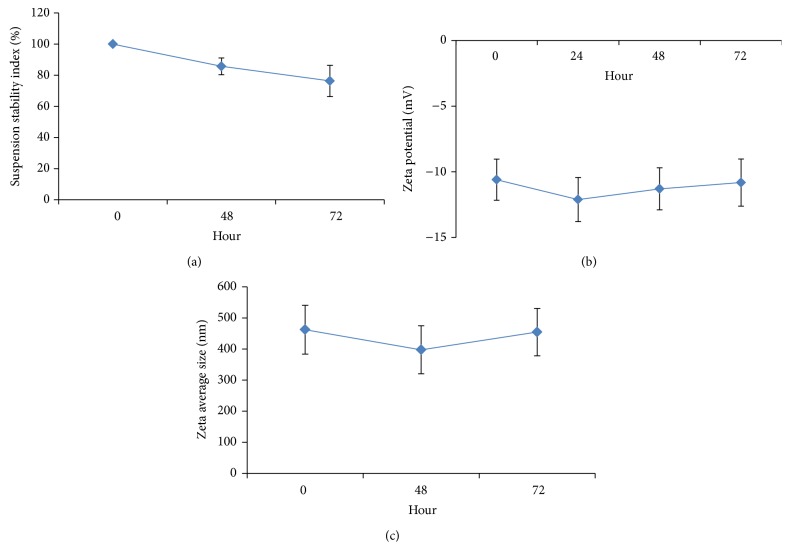
MWCNT dispersion state. (a) Suspension stability index. The suspension stability index of the MWCNTs at 100 mg/L (100 *μ*g/mL) is expressed as the % of the initial absorbance (*λ* = 550 nm) at time 0 for MWCNTs suspended in water for 42 and 72 h after dispersion. The spot shows the average stability index measured at every 0, 48, and 72 h. (b) and (c) Zeta-potential value and Zeta average size: the Z-potential values and Zeta average size measure at every at every 0, 24, 48, and 72 h.

According to the manufacturer's product datasheet, the MWCNTs were produced via a catalytic chemical vapor deposition process and purified to over 90%, with the major impurities being the metals Al (~2.3 wt%), Fe (~0.7 wt%), and Mo (~0.15 wt%). They had diameters of 10–15 nm, an average length of 20 *μ*m, and a bulk density of 0.035 g/mL (Figure 1). The particle size and Zeta potential of the MWCNTs were characterized using dynamic light scattering (DLS; Zetasizer nanoseries, nano-zs90, Malvern Instruments, Worcestershire, UK). To analyze the suspension stability index of the MWCNTs, the ultraviolet- (UV-) absorbance (*λ* = 550 nm) was measured using a UV-vis spectrophotometer (Lambda 25, PerkinElmer, MA, USA). The suspension stability index of the MWCNTs at 100 mg/L (100 *μ*g/mL) is expressed as the % of the initial absorbance (*λ* = 550 nm) at time 0 for MWCNTs suspended in water for 42 and 72 h after dispersion (Figure 2). The morphology of the MWCNTs was investigated using scanning electron microscopy (SEM; JEOL JSM 6700F, JEOL, Tokyo, Japan) (Figure 1(a)) and transmission electron microscopy (TEM; FEI Tecnai 20, 200 kV, FEI, Hillsboro, OR, USA) (Figure 1(b)).

### 2.3. Fish Care

*Oryzias latipes* were received from the aquarium of the Environmental Toxicology Center of Korea Institute of Toxicology, Jin-Ju, Gyeongsangnam Province, South Korea (5-month-old adult male and female fish; length = 2.9 ± 0.36 cm, weight = 0.27 ± 0.11 g). All fish were acclimatized in the laboratory for 1 month prior to the start of the study. Every morning and evening, they were fed 1% of their total weight using a commercial feed (tetramin tropical flake, Tetra Co., Melle, Germany). The acclimatization tank was filled with dechlorinated tap water and the tank water was changed once every 2 days. The photoperiod was a 12 h light/12 h dark cycle. Other conditions were maintained as follows: water temperature = 22 ± 1°C, dissolved oxygen = >80%, and pH = 6.8–7.4.

### 2.4. Oxidative Stress Assay

**Figure 3:**
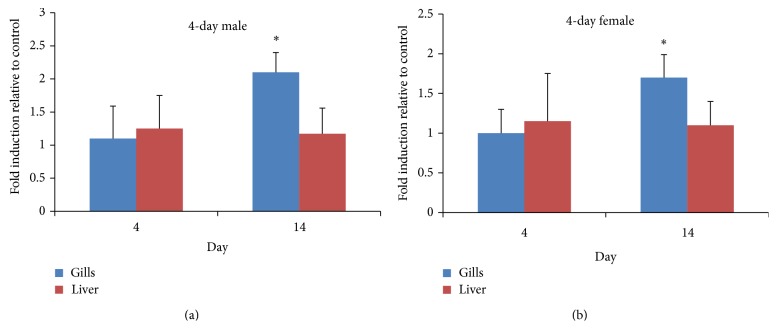
Intracellular ROS level. (a) Intracellular ROS induction level relative to control in male* O. latipes* exposed to 100 mg/L MWCNT. (b) Intracellular ROS induction level relative to control in female* O. latipes* exposed to 100 mg/L MWCNT. Columns and error bars represent the mean ± SD. Asterisk indicates values that are significantly higher than control values (^*^ *P* < 0.05;* t*-test, *n* = 5).

The intracellular reactive oxygen species (ROS) was measured with dichlorofluorescein-diacetate method. A DCF-DA stock solution of 10 mM (in DMSO) was diluted 500-fold in Hank's balanced salt solution (HBSS; ThermoFisher scientific, Inc., Waltham, MA, USA) to yield a 20 *μ*M working solution. A 10-mg sample of each tissue was homogenized with TissueLyser LT (Qiagen) in PRO-PREP protein (Intron Biotechnology Co., Seoul, Republic of Korea) extraction solution, according to the manufacturers' instructions. Following centrifugation at 13,000 ×g, protein in the supernatant was quantified by the Bradford assay. The 20 *μ*g protein from each tissue lysate was mixed with DCF-DA working solution and the mixture was incubated for 3 hours at 37°C in the dark. Fluorescence of the samples was monitored at an excitation wavelength of 485 nm and an emission wavelength of 538 nm using fluorescence plate reader (Synergy H1 hybrid reader, BioTek instruments, Inc., Winooski, VT, USA). The result is shown in Figure 3.

### 2.5. Exposure Design

In this study, we select 100 mg/L (100 *μ*g/mL) MWCNT as exposure concentration. The reason to select this concentration is that in preliminary test, statistically significant* Cas3*,* Cas8*, and* Cas9* gene induction relative to control was confirmed in 100 mg/L MWCNT exposure. There was no significant gene induction in 1 and 10 mg/L MWCNT exposure. For the waterborne exposure, 100 mg MWCNT was dispersed in 1 L distilled water containing 0.01% HCO-40 lipocol through sonication (56% output, sonication for 10 min, followed by a 5 sec pause, repeat three times) with sonicator (GE 70T fisher scientific, Pittsburgh, PA,USA). After dispersion, each 1 L dispersed MWCNT solution was pooled into 7 L tank, and 25* O. latipes* were administrated into it. The experimental tank was changed every 3 days for 14 days. And every 0 and 3 days, Zeta potential, Zeta average size, and UV absorbance at 550 nm for each treatment solution were measured using DLS and UV-vis spectrophotometer (Figure 2). And another 25 fish were exposed to solvent control (HCO-40) 7 L tank containing 0.01% HCO-40 for 14 days. We decapitated 5 fish from the acclimatization tank just before specimens were introduced into the treatment tank and used their liver and gills as the 0-day sample; that is, a calibrator that represents the amount of mRNA expressed at time zero in the control [43] (Livak and Schmittgen 2001). After exposure, at 4, 14 days, 7* O. latipes* were decapitated. Liver, intestine, and gills were excised and extracted tissues were immediately immersed in RNA* later* solution (Qiagen, Valencia, CA, USA) according to the manufacturer's recommendations.

### 2.6. Primer Design

*Beta-actin, Cas3, Cas8, Cas9, p53, metallothionein (MT), catalase, glutathione-s-transferase (GST)* gene primer sequences were designed using the GenBank database described in Table 1. For each primer, application efficiency test was performed; the slopes of each primer were within 95–105%.

### 2.7. RNA Extraction and Real-Time PCR

Liver, gills, and intestine tissues preserved in RNA*later* were homogenized with TissueLyser II (Qiagen) and total RNA was extracted using an RNeasy mini prep kit (Qiagen) according to the manufacturer's procedures. Total RNA quantity was estimated using a microspectrophotometer (Bio-prince SD 2000, China) at 260 nm. The values for 260/280 nm were 2.01 ± 0.05 (average ± SD). To check the quality of the total RNA estimates, the bands for 28S and 18S ribosomal RNA from all samples were confirmed by 1% agarose gel electrophoresis. A 1 *μ*g RNA sample was reverse transcribed into cDNA using M-MLV reverse transcriptase (Promega Corp., Madison, WI, USA). Then 10 *μ*L Brilliant III ultra-fast SYBR Green QPCR Mix (Agilent Technologies, Santa Clara, CA, USA), 9 *μ*L cDNA (diluted to 1/50 with DW), and 5 pmol 1 *μ*L primer were mixed and reacted in an Mx3000P qRT-PCR system (Stratagene, La Jolla, CA, USA). The *β*-actin housekeeping gene was used as a reference to normalize the expression levels. The PCR reaction cycle was conducted as follows: 94°C for 3 min, followed by 40 cycles at 94°C for 20 s, 60°C for 15 s, and 72°C for 15 s. Melting curve analysis was performed to check the specific product. Real-time PCR data were obtained as threshold cycle (*C*_*T*_) values, and fold changes for relative gene expression to the calibrator were determined using the 2^−ΔΔCT^ method [43] (Livak and Schmittgen 2001). There was no significant change in beta-actin mRNA expression during 14 days (one-way ANOVA, *P* < 0.05).

### 2.8. Statistical Analysis

The* t*-tests were used to detect significant effects between 100 mg/L MWCNT exposure and the corresponding control groups. A one-way ANOVA followed by Tukey's test was used to detect significant difference among treated groups. Data were expressed as means ± SD. All data were analyzed using Minitab for windows software (Minitab Inc., State College, PA, USA). Prior to the ANOVA and *t*-test, normality test for data was performed with Anderson-Darling test. *F*-test and Levene's tests were carried out to evaluate homogeneity of variance. A *P* value less than 0.05 were considered significant.

## 3. Results

### 3.1. MWCNT Characterization

As seen in the TEM and SEM micrographs, MWCNTs, not some other form of carbon nanoparticle or amorphous carbon, were present (Figure 1). To analyze the dispersed state of the MWCNTs, we observed the stability index, Zeta potential, and Zeta average size (Figure 2). During exposure period, the suspension was maintained at over 73% and there was no significant alteration in the Zeta potential or Zeta average size during exposure. The tank water in which the MWCNTs were dispersed was replaced every 3 days. Figure 2 showed the dispersal status of the MWCNTs in water containing 0.01% HCO-40. Therefore, any toxicological outcomes identified in this study can be attributed to exposure to MWCNTs.

### 3.2. Oxidative and Metal Stress

**Figure 4:**
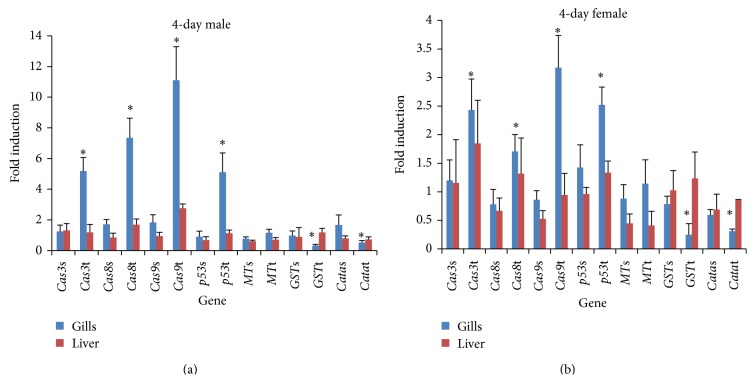
mRNA induction at 4 days. (a) Gene expression pattern of male* O. latipes* exposed to 100 mg/L MWCNT and solvent control. (b) Gene expression pattern of female* O. latipes* exposed to 100 mg/L MWCNT and solvent control. The initials S and T followed by gene name reveal the solvent control group and MWCNT treated group, respectively. For example,* Cas3*t is the* Cas3* expression pattern of MWCNT treated group and* Cas3*s is the pattern of solvent control group. Columns and error bars represent the mean ± SD. Asterisk indicates values that are significantly higher than control values (^*^ *P* < 0.05;* t*-test, *n* = 7).

**Figure 5:**
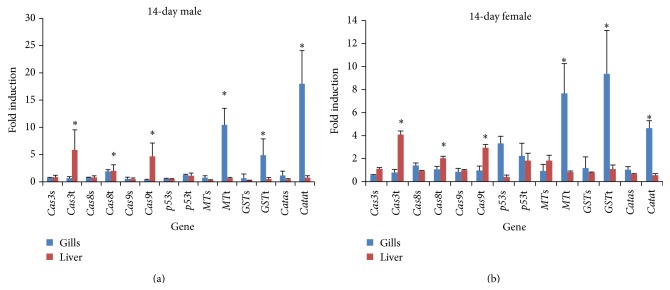
mRNA induction at 14 days. (a) Gene expression pattern of male* O. latipes* exposed to 100 mg/L MWCNT and solvent control. (b) Gene expression pattern of female* O. latipes* exposed to 100 mg/L MWCNT and solvent control. The initials S and T followed by gene name reveal the solvent control group and MWCNT treated group, respectively. For example,* Cas3*t is the* Cas3* expression pattern of MWCNT treated group and* Cas3*s is the pattern of solvent control group. Columns and error bars represent the mean ± SD. Asterisk indicates values that are significantly higher than control values (^*^ *P* < 0.05;* t*-test, *n* = 7).

The oxidative stresses induced by MWCNTs were investigated by examining intracellular ROS and* GST* and* CAT* gene expression. Significant antioxidant gene induction was observed at 14 days of MWCNT exposure (Figure 5). In addition, at 4 days, significant reductions in* CAT* and* GST* expression in the gills were observed (Figure 4). Relative to the control, the* GST* and* CAT* reduction in males at 4 days was ca. three- and twofold, respectively, and, at 14 days, the* GST* and* CAT* induction was roughly 7- and 17-fold (Figures 4 and 5). In females the respective* GST* and* CAT* reduction was ca. 2.3- and 2-fold at 4 days and* GST* and* CAT* induction was 9- and 4-fold at 14 days. Significant* MT* gene expression was induced 10- and 7-fold relative to the control at 14 days in the gills of males and females, respectively.

Examining the gender differences in the gills, at 4 days,* CAT* and* GST* expression in the gills was reduced significantly, while* MT* expression was unchanged, although only the reduction in* CAT* differed significantly between males and females. At 14 days, the average levels of induction of* MT* and* CAT* were higher in males, while* GST* expression was higher in females, although the difference was significant only for* CAT* (one-way ANOVA, *P* < 0.05).

Differences in expression were observed among the gills, liver, and intestine. At 4 days,* GST* and* CAT* expression were reduced significantly relative to the controls, while in the liver no significant reduction was found. At 14 days, in the gills,* GST*,* CAT*, and* MT* expression were induced significantly relative to the control, while in the liver there was no significant induction. In the intestine, no significant expression of oxidative stress genes was observed.

### 3.3. Apoptosis Gene Expression

MWCNT-induced apoptosis was investigated by examining* p53*,* Cas3*,* Cas8*, and* Cas9* gene expression. At 4 days,* p53*,* Cas3*,* Cas8*, and* Cas9* expression was increased significantly relative to the controls and was found with 100 mg/L MWCNT exposure in the gills of both females and males (Figure 4). The expression of the* p53*,* Cas3*,* Cas8*, and* Cas9* genes in the gills of males was increased 5-, 5-, 7-, and 11-fold, respectively, while that in the gills of females was increased 2-, 2-, 2.5-, and 4-fold, respectively. At 14 days, significant induction of* Cas3*,* Cas8*, and* Cas9* gene expression relative to the controls was found in the livers of both females and males (Figure 5). The expression of* Cas3*,* Cas8*, and* Cas9* was increased 5-, 2.5-, and 7-fold, respectively, in males and 4-, 2-, and 3-fold in females.

There was a gender difference in apoptosis gene expression. The induction of* Cas3*,* Cas8*,* Cas9*, and* p53* expression relative to the controls was significantly higher in males than females. At 14 days, the average fold induction relative to the controls in the liver was higher in males than females, but the difference between males and females was significant only for* Cas9*.

Differences in the induction of apoptosis gene were found among the gills, liver, and intestine. At 4 days, significant induction of expression relative to the controls was found only in the gills, thus not the liver, while at 14 days significant induction of expression was found only in the liver, not the gills.* Cas8* and* Cas9* expression was induced to a significantly greater degree in the gills than the liver.

## 4. Discussions

Scanning and transmission electron microscopy showed MWCNTs, and not some other form of carbon nanoparticle or amorphous carbon (Figure 1). There was no significant alteration of hydrodynamic size, Zeta-potential, and stability index during MWCNT exposure. Therefore, any toxicological outcomes identified in this study are attributable to MWCNT exposure.

Intracellular ROS generation was measured using a dichlorofluorescein-diacetate method. No significant ROS induction relative to the controls was observed in any tissue at 4 days in which a decrease in antioxidant gene expression was found. These results are contradictory. Some studies revealed ROS induction caused by carbon nanotubes [8, 9, 14, 16, 44–46], while others failed to detect ROS* in vitro* or* in vivo* [39, 47–50].* Daphnia magna* exposed to MWCNT did not cause ROS induction [39]. Kagan et al. [51] demonstrated that neither purified (0.23 wt. % of iron) nor nonpurified CNTs (26 wt. % of iron) led to intracellular production of superoxide radicals in RAW 264.7 macrophages, while CNTs generated hydroxyl radicals in zymosan-stimulated RAW 264.7 macrophages. Any metal impurities from MWCNT can also induce ROS [29]. Nevertheless, at 4 days, no such ROS induction was observed. Fenoglio et al. [52] stated that CNTs could scavenge hydroxyl radicals produced by the Fenton reaction. Furthermore, after 4 days of MWCNT exposure, no induction of* MT* expression was evident. Together, these results suggest that metal impurities did not participate in ROS production.

At 14 days, ROS induction was observed with simultaneous significant induction of antioxidant and* MT* gene expression in the gills. Previous studies showed the ROS can be induced by metal impurities from carbon nanotubes. Ultrasonication used in many studies including this work to disperse carbon nanotube in water increased bioavailability of metal impurities [53]. The ROS production induced by the metal impurities (especially iron) from carbon nanotube was observed [29, 37, 51]. The bioaccumulation of metals, such as iron in fish liver and gills, has been reported [1, 54]. So, we postulate that after 4 days of MWCNT exposure, insufficient metal impurities have not been accumulated to the necessary threshold level and MWCNTs scavenge any hydroxyl radicals produced by the metal impurities, likely repressing antioxidant gene expression and ROS increase. By 14 days, however, sufficient metal impurities might be accumulated to overcome the antioxidant depletion and MWCNT scavenging activity, leading to significantly increased* MT* and antioxidant gene expression. Together, these results suggest that the significant increase in antioxidant and* MT* gene expression at 14 days may be due to the effects of metal impurities as well as MWCNTs.

The expression levels of antioxidant genes were reduced significantly relative to the controls at 4 days. This result was observed in other studies in which rodents or cells were exposed to MWCNTs [9, 11, 14, 30, 46, 55, 56]. Those studies mentioned that the prooxidant effects of engineered nanomaterials were mediated by depletion of antioxidants. Such perturbations of the normal redox state contribute to peroxide and free radical production, which has adverse effects on proteins, lipids, and DNA [30]. Reduced* GST*,* CAT*, and* SOD* gene expression and decreased protein activity were also found in studies in which fish were exposed to Ag and ZnO nanoparticles [57–59]. Thus, it is postulated that antioxidant depletion observed in this work might be caused specifically by nanosize particles irrespective of the type of nanomaterial as well as the CNT capacity which scavenge ROS.

At 4 days, significant induction of apoptosis gene (*p53*,* Cas3*,* Cas8*, and* Cas9*) expression in the gills was observed relative to the controls on exposure to 100 mg/L MWCNTs, while there was no significant induction of expression in the liver or intestine. The proteases encoded by* Cas3*,* Cas8*, and* Cas9* play pivotal roles in the apoptosis pathway. In addition,* p53* plays an important role in cell cycle control and apoptosis by activating various proapoptotic genes, such as* Bax* [60]. In previous studies,* Cas3*,* Cas8*, and* Cas9* expression was found to be an indicator of MWCNT-induced apoptosis [9, 13, 15]. Previous studies also demonstrated that MWCNT induced* p53*-dependent apoptosis [9, 61]. At 14 days, no significant induction of apoptosis gene expression was found in the gills. However,* Cas3*,* Cas8*, and* Cas9* expression increased significantly in liver. The fold induction was lower than that in the gills at 4 days. Therefore, ca. 14 days are needed for MWCNT-induced apoptosis to affect the liver. Several studies showed that MWCNT could translocate to other organs of the body through barrier organ such as lung, causing systemic side effects [62, 63]. Thus, it is likely that translocation of MWCNT can be the reason of apoptosis occurrence in liver. In addition, metal impurities can also cause ROS-dependent apoptosis. However, since there was no significant induction of the expression of* MT* or antioxidant genes in the liver, whether metal impurities caused the apoptosis gene induction in the liver at 14 days is unclear. Overall, these results suggest that MWCNT dispersed in water can cause apoptosis occurrence in gill and liver of fish at different exposure period.

In this work, significant gender differences were found. Previous studies demonstrated that estradiol or endocrine-disruptor chemicals could induce the expression of antioxidant genes, such as* GST*, but reduce* CAT* and* MT* expression [24, 28, 64]. At 14 days, the* GST* induction relative to the controls was higher in females than males, albeit not significantly so, while the induction of* MT* and* CAT* expression was significantly higher in males. At 4 days, antioxidant gene expression was reduced relative to the controls. In males,* CAT* expression was reduced to a significantly extent than in females. This result differs from previous reports. The MWCNT-induced expression of apoptosis gene differed according to gender. The fold induction of* Cas3*,* Cas8*,* Cas9*, and* p53* expression relative to the controls was significantly higher in male than female fish at 4 days after exposure to 100 mg/L MWCNT. Previous studies demonstrated that estrogen repressed p53-dependent apoptosis [23, 27]. Bailey et al. [27] reported that a small set of proapoptotic* p53* target genes is also targeted by the estrogen receptor (ER).

The differences in apoptosis and antioxidant gene expression among tissues were analyzed. Only gills showed significantly increased gene expression at 4 days. As mentioned above, the mechanisms of MWCNT-induced apoptosis and antioxidant depletion are unclear. MWCNTs can lead to frustrated phagocytosis, which would results in release of superoxide produced in macrophage phagosomes into intra- and extracellular space [31, 32, 65, 66]. Previously, several studies showed that superoxide fluxes can cross the endothelial cell plasma membrane via the chloride channel (CLC-3), causing intracellular ROS stress and apoptosis [13, 33, 34]. CLC-3 can be induced by specific environmental stresses, such as anion alteration, at higher levels in gills than in the liver or intestine [67, 68]. Therefore, the influence caused by MWCNT exposure could lead to the expression of CLC-3 channels through which superoxide translocates, to a greater extent in the gills than in the liver or intestine. Further investigation of tissue differences in MWCNT-induced ROS and apoptosis production is needed.

In the histological study, no apoptosis or inflammation was found after MWCNT exposure for 21 days (Suppl. Figure 1) (see Supplementary Material available online at http://dx.doi.org/10.1155/2014/485343). van der Oost et al. [1] reported that a molecular-level response to chemicals occurs before tissue-level effects are evident. Therefore, it is necessary to assess the correlation between the biological responses to MWCNTs at the molecular and tissue levels by means of tests involving prolonged MWCNT exposure.

In summary, this study showed that MWCNT exposure could cause antioxidant depletion and p53-dependent apoptosis. The expression of relevant genes was influenced by exposure duration and differed according to gender and tissue type.

## Supplementary Material

In Supplementary figure 1-C, it is apparent that this is a typical section stained by hematoxylin and eosin illustrating common features of the luminal and mural elements of the intestine. In supplementary figure 1-D, apoptotic hepatocytes which have a structure that can be recognized as large cytoplasmic vacuoles are not observed. In supplementary figure 1-E and F, dark materials which are not shown in control group (Supplementary Figure 1-B and -C) is considered as MWCNT. At the lower right hand of this field of supplementary figure 1-E, exocrine pancreatic cells are seen. There was no inflammatory and apoptotic cells in both control and treated group.

## Figures and Tables

**Table 1:** Primers used in this work.

Gene	Accession number	Sequence	Product size (base pair)
B-Actin-F	S74868.1	TCC ACC TTC CAG CAG ATG TG	75
B-Actin-R		AGC ATT TGC GGT GGA CG AT	

Caspase 3-F	NM_001104670.1	TGG GTC CTC GTA ACG GTA CA	131
Caspase 3-R		TGG ACA TTT GGC GAA ACA GC	

Caspase 8-F	NM_001104788.1	ACC GAG CCC CTA GCT TGA TA	108
Caspase 8-R		GCA TCC CCT TTC ACT TCC GA	

Caspase 9-F	XM_004070317.1	CTG GCG ACG TAC AGT CTC AG	81
Caspase 9-R		AGT TGC AGC ATG TTC CTC GA	

GST-F	XM_004085912.1	ACC TGC GAT CAC ACT GTT CA	77
GST-R		TTT GGA GAC TTC AGA GCC CA	

MT-F	NM_001104785.1	CCG ACT CGA CTC TGA CAG AC	88
MT-R		CAG TCG CAG GGG TCC ATT AT	

Catalase-F	XM_004069460.1	GCG GTA CAA CAG CGC AGA TG	170
Catalase-R		GGA TGG ACG GCC TTC AAG TT	

P53-F	U57306.1	GTT CGT AGC TTC CCG GGT AG	85
P53-R		GAT TGA GCC AGT TCC CAC CA	

MT: metallothionein; GST: glutathione-s-transferase; B-actin: beta-actin.
