# Supplementary figures and images for: Multiwall Carbon Nanotube-Induced Apoptosis and Antioxidant Gene Expression in the Gills, Liver, and Intestine of Oryzias latipes
Source: Biomed Res Int. 2015 Jun 3;2015:485343. doi: 10.1155/2015/485343 (PMC4469764; doi:10.1155/2015/485343)

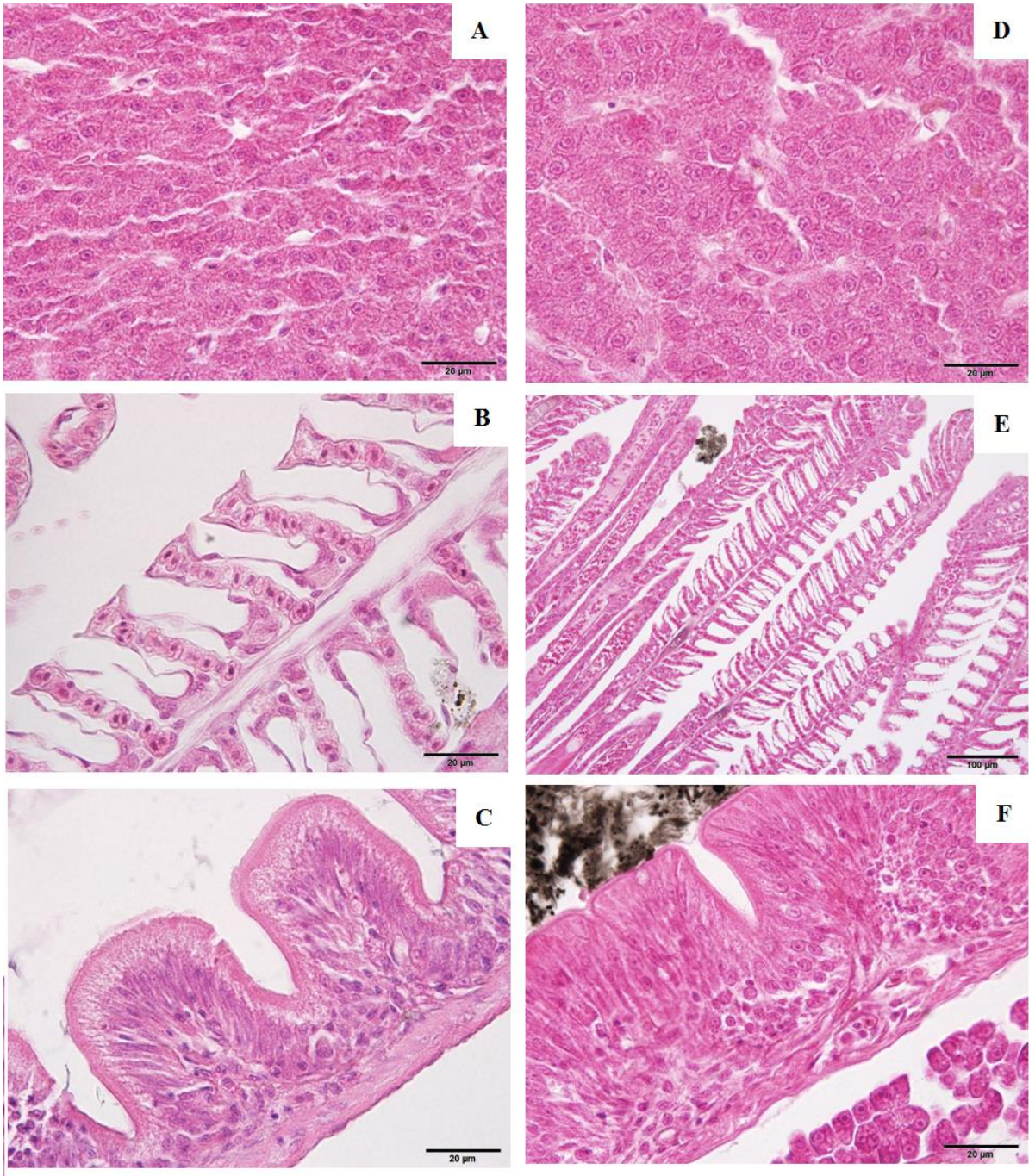

Supplement: Supplementary file 1 — In Supplementary figure 1-C, it is apparent that this is a typical section stained by hematoxylin and eosin illustrating common features of the luminal and mural elements of the intestine. In supplementary figure 1-D, apoptotic hepatocytes which have a structure that can be recognized as large cytoplasmic vacuoles are not observed. In supplementary figure 1-E and F, dark materials which are not shown in control group (Supplementary Figure 1-B and -C) is considered as MWCNT. At the lower right hand of this field of supplementary figure 1-E, exocrine pancreatic cells are seen. There was no inflammatory and apoptotic cells in both control and treated group. [file 485343.f1.pdf]
